# CCDC106 promotes non-small cell lung cancer cell proliferation

**DOI:** 10.18632/oncotarget.15792

**Published:** 2017-03-01

**Authors:** Xiupeng Zhang, Qin Zheng, Chen Wang, Haijing Zhou, Guiyang Jiang, Yuan Miao, Yong Zhang, Yang Liu, Qingchang Li, Xueshan Qiu, Enhua Wang

**Affiliations:** ^1^ Department of Pathology, College of Basic Medical Sciences, China Medical University, Shenyang, China; ^2^ Department of Pathology, First Affiliated Hospital, China Medical University, Shenyang, China; ^3^ Department of Pathology, Cancer Hospital of China Medical University, Shenyang, China

**Keywords:** CCDC106, lung cancer, AKT signaling, cyclin A2, cyclin B1

## Abstract

Coiled-coil domain containing (CCDC) family members enhance tumor cell proliferation, and high CCDC protein levels correlate with unfavorable prognoses. Limited research demonstrated that CCDC106 may promote the degradation of p53/TP53 protein and inhibit its transactivity. The present study demonstrated that CCDC106 expression correlates with advanced TNM stage (*P* = 0.008), positive regional lymph node metastasis (*P* < 0.001), and poor overall survival (*P* < 0.001) in 183 non-small cell lung cancer cases. A549 and H1299 cells were selected as representative of CCDC106-low and CCDC106-high expressing cell lines, respectively. CCDC106 overexpression promoted A549 cell proliferation and xenograft tumor growth in nude mice, while siRNA-mediated CCDC106 knockdown inhibited H1299 cell proliferation. CCDC106 promoted AKT phosphorylation and upregulated the cell cycle-regulating proteins Cyclin A2 and Cyclin B1. Cell proliferation promoted by CCDC106 via Cyclin A2 and Cyclin B1 was rescued by treatment with the AKT inhibitor, LY294002. Our studies revealed that CCDC106 is associated with non-small cell lung cancer progression and unfavorable prognosis. CCDC106 enhanced Cyclin A2 and Cyclin B1 expression and promoted A549 and H1299 cell proliferation, which depended on AKT signaling. These results suggest that CCDC106 may be a novel target for lung cancer treatment.

## INTRODUCTION

Coiled-coil domain containing 106 protein (CCDC106), previously called HSU79303 [1], may promote p53 degradation through direct interaction in Hela cells [2]. *TP53* is a tumor suppressor and is responsible for DNA damage and cell cycle regulation [3–6]. P53 dysfunction promotes cancer development and progression, and selective elimination of p53 defective cancer cells represents an ideal therapeutic strategy [7–8]. We hypothesized that CCDC106 is involved in human cancer progression and cell cycle regulation. Previous studies indicated that other CCDC family members enhance tumor cell proliferation, and high protein levels correlate with unfavorable prognosis [9].

In this study, we explored CCDC106 expression patterns and subcellular distributions in both lung cancer tissues and cell lines. We found that CCDC106 overexpression in non-small cell lung cancer (NSCLC) tissues correlated with advanced TNM stage, positive regional lymph node metastasis, and poor overall survival. *TP53* wild-type cell line, A549, and *TP53* null cell line, H1299, were used to analyze whether CCDC106 regulated lung cancer cell proliferation through p53 signaling. We found that CCDC106 increased Cyclin A2 and Cyclin B1 expression, promoting cell proliferation *in vitro* and *in vivo* through AKT pathway activation.

## RESULTS

### CCDC106 overexpression correlates with unfavorable NSCLC patient prognosis

**Figure 1:**
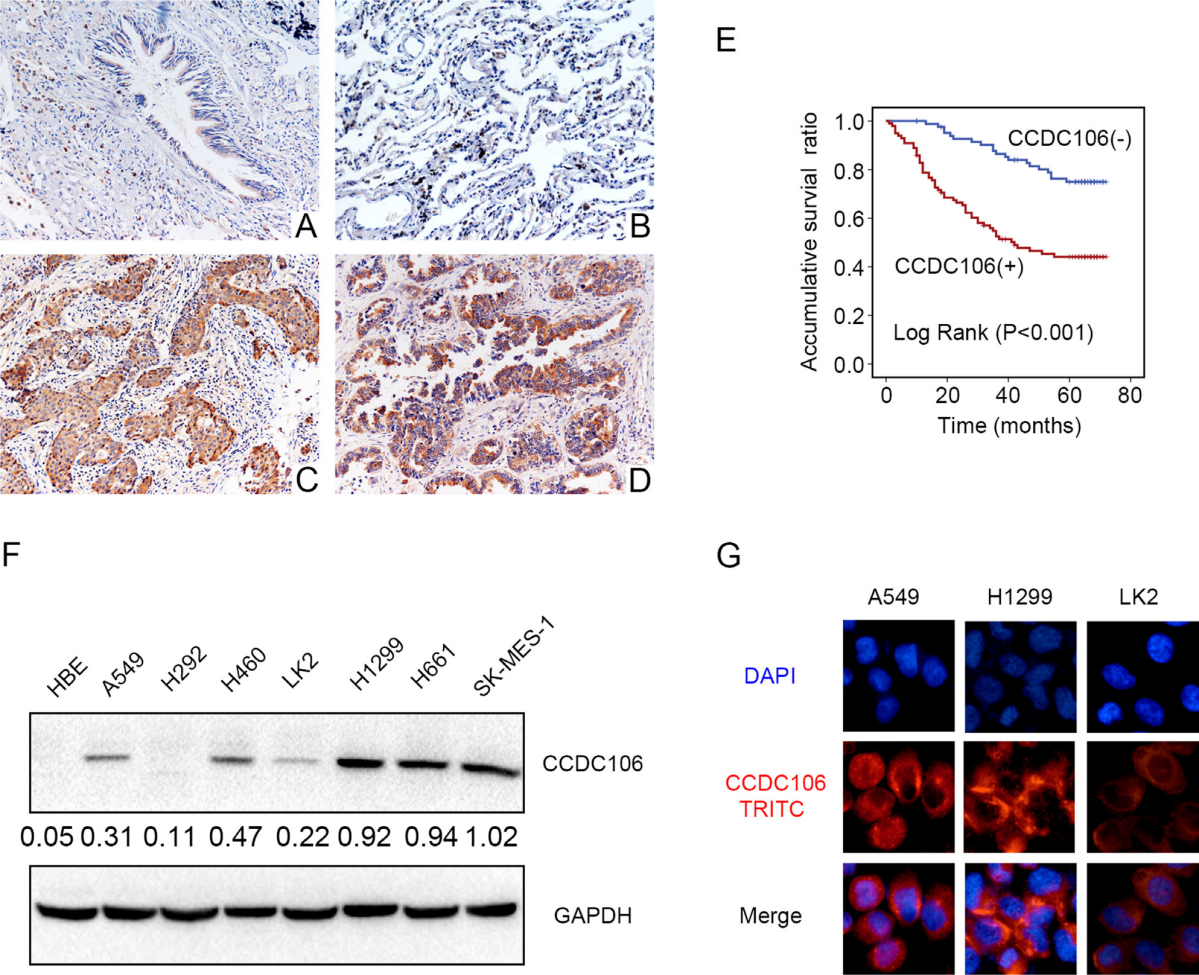
CCDC106 expression in NSCLC specimens and cell lines Negative or weak CCDC106 expression in normal bronchial (**A**) and alveolar epithelial cells (**B**) Positive CCDC106 expression in the cytoplasm of lung squamous cell carcinoma (**C**) and adenocarcinoma cells (**D**) Kaplan–Meier survival analysis revealed that CCDC106-positive patient overall survival was reduced compared to those without CCDC106 expression (**E**) CCDC106 expression in HBE cells was lower than in most NSCLC cell lines (except H292) (**F**) and was localized to the cytoplasm in A549, H1299 and LK2 cells (**G**).

We performed immunohistochemical (IHC) staining in 183 NSCLC tissue samples and 58 peritumoral lung tissue samples to evaluate the expression pattern of CCDC106. Compared with peritumoral lung tissues (Figure 1A–1B), CCDC106 exhibited higher cytosolic expression (54.1% vs 20.7%, *P* < 0.001) in NSCLC samples (Figure 1C–1D). Positive cytosolic expression was correlated with advanced TNM stage (*P* = 0.008) and positive lymph node metastasis (*P* < 0.001, Table 1). However, there was no association between CCDC106 overexpression and patient sex, age, tumor histological type, or histological differentiation (Table 1). Kaplan-Meier analysis showed that CCDC106-positive patient overall survival (43.539 ± 2.790 months) was shorter than that of negative or weak CCDC106-expressing patients (63.021 ± 1.895 months, *P* < 0.001, Figure 1E). Cox univariate and multivariate analysis revealed that advanced TNM stage and cytosolic CCDC106 overexpression (*P* < 0.001 and *P* = 0.001, respectively; Table 2) were independent prognostic factors in NSCLC patients.

**Table 1:** Correlation of CCDC106 expression with clinicopathological features in 183NSCLC cases

Clinicopathological factors	*N*	Positive	Negative	χ2	*P*
Age (years)					
< 61	72	43	29	1.512	0.229
≥ 61	111	56	55		
Gender					
Male	112	64	48	1.078	0.361
Female	71	35	36		
Histological type					
Squamous cell carcinoma	66	32	34	3.997	0.136
Adenocarcinoma	115	67	48		
Large cell carcinoma	2	0	2		
Differentiation					
Well	75	37	38	1.162	0.295
Moderate+Poor	108	62	46		
TNM classification					
I+II	120	56	64	7.753	0.008
III	63	43	20		
Lymph node metastasis					
Positive	86	59	27	13.749	< 0.001
Negative	97	40	57		

**Table 2:** Summary of cox univariate and multivariate regression analysis of the association between clinicopathological features and overall survival in 183 cases of non-small cell lung cancer (NSCLC)

Clinicopathological feature	Hazard ratio (95% CI)	*P*
**Univariate analysis**		
Age older than 61	0.903 (0.565–1.443)	0.669
Gender: male	1.010 (0.632–1.614)	0.966
Histological type : Adenocarcinoma	1.552 (0.967–2.492)	0.069
Poor differentiation	1.651 (1.012–2.691)	0.044
High TNM classification	4.532 (2.811–7.307)	< 0.001
Positive lymph node metastasis	5.001 (2.949–8.481)	< 0.001
Positive CCDC106 expression	3.223 (1.922–5.403)	< 0.001
**Multivariate analysis**		
High TNM classification	2.301 (1.201–4.407)	0.012
Positive CCDC106 expression	2.466 (1.432–4.249)	0.001

We next performed western blot (WB) and immunofluorescence analyses in seven NSCLC cell lines and a normal bronchial epithelial cell line (HBE) to detect CCDC106 and assess its subcellular localization. Compared with HBE cells lacking CCDC106 expression, CCDC106 was detected in 6/7 NSCLC cell lines, except in H292 cells (Figure 1F). Immunofluorescence results indicated that CCDC106 localized in the cytoplasm of A549, H1299, and LK2 cells (Figure 1G).

### CCDC106 enhanced NSCLC proliferation *in vitro* and *in vivo*

**Figure 2:**
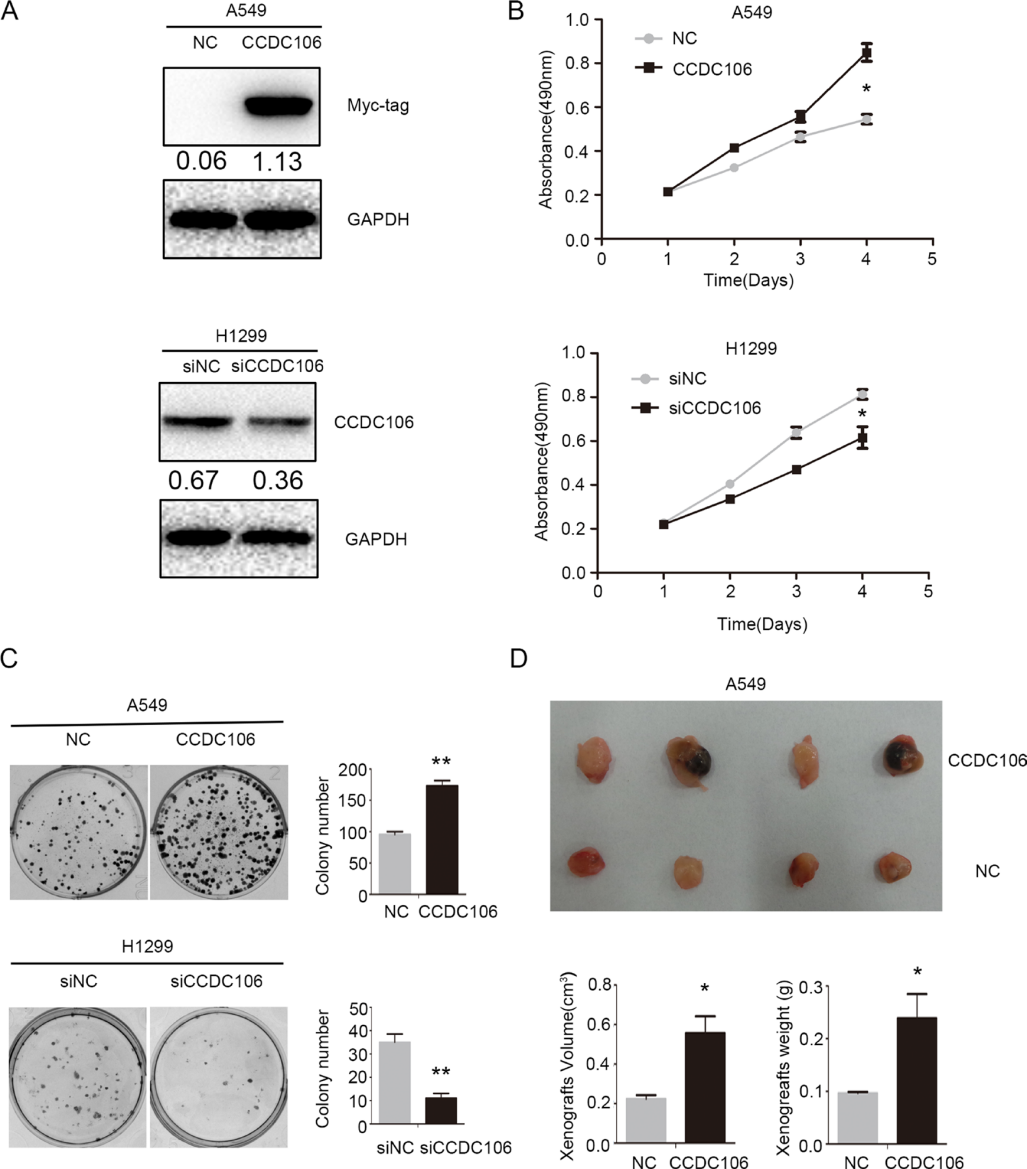
CCDC106 enhanced NSCLC cell proliferation *in vitro* and *in vivo* Transfection efficiency after overexpressing CCDC106 in A549 cells and silencing CCDC106 in H1299 cells, as tested by WB (**A**) CCDC106 overexpression enhanced A549 cell growth (**B**) and colony formation (**C**) while knockdown reduced H1299 cell growth (B) and colony formation C. Average tumor volumes and weights were higher in mice subcutaneously injected with A549-CCDC106 ^(+)^ cells compared to controls (**D**). *P < 0.05, **P < 0.001.

Based on previous data (Figure 1F) and *TP53* status, A549 and H1299 cells were selected as representative of CCDC106-low and CCDC106-high cell lines, respectively. The transfection efficiencies of pCMV-CCDC106 or CCDC106-siRNA were confirmed by WB in A549 and H1299 cells (Figure 2A). As measured by MTT assay, CCDC106 overexpression enhanced A549 cell proliferation. Conversely, CCDC106 knockdown decreased H1299 cell proliferation compared to controls (Figure 2B). Consistent with MTT assay results, CCDC106 overexpression in A549 cells increase foci numbers and sizes. However, CCDC106 knockdown in H1299 cells decreased foci numbers and sizes (Figure 2C).

We subcutaneously injected CCDC106-transfected A549 cells (A549-CCDC106 ^(+)^) or control cells into nude mice axillae. Average tumor volumes (0.557 ± 0.170 cm^3^) and weights (0.239 ± 0.092 g) in the A549-CCDC106 ^(+)^ group were higher than in the control group (0.224±0.037 cm^3^ and 0.096 ± 0.005 g, respectively; Figure 2D).

### CCDC106 upregulated Cyclin A2 and Cyclin B1 expression

**Figure 3:**
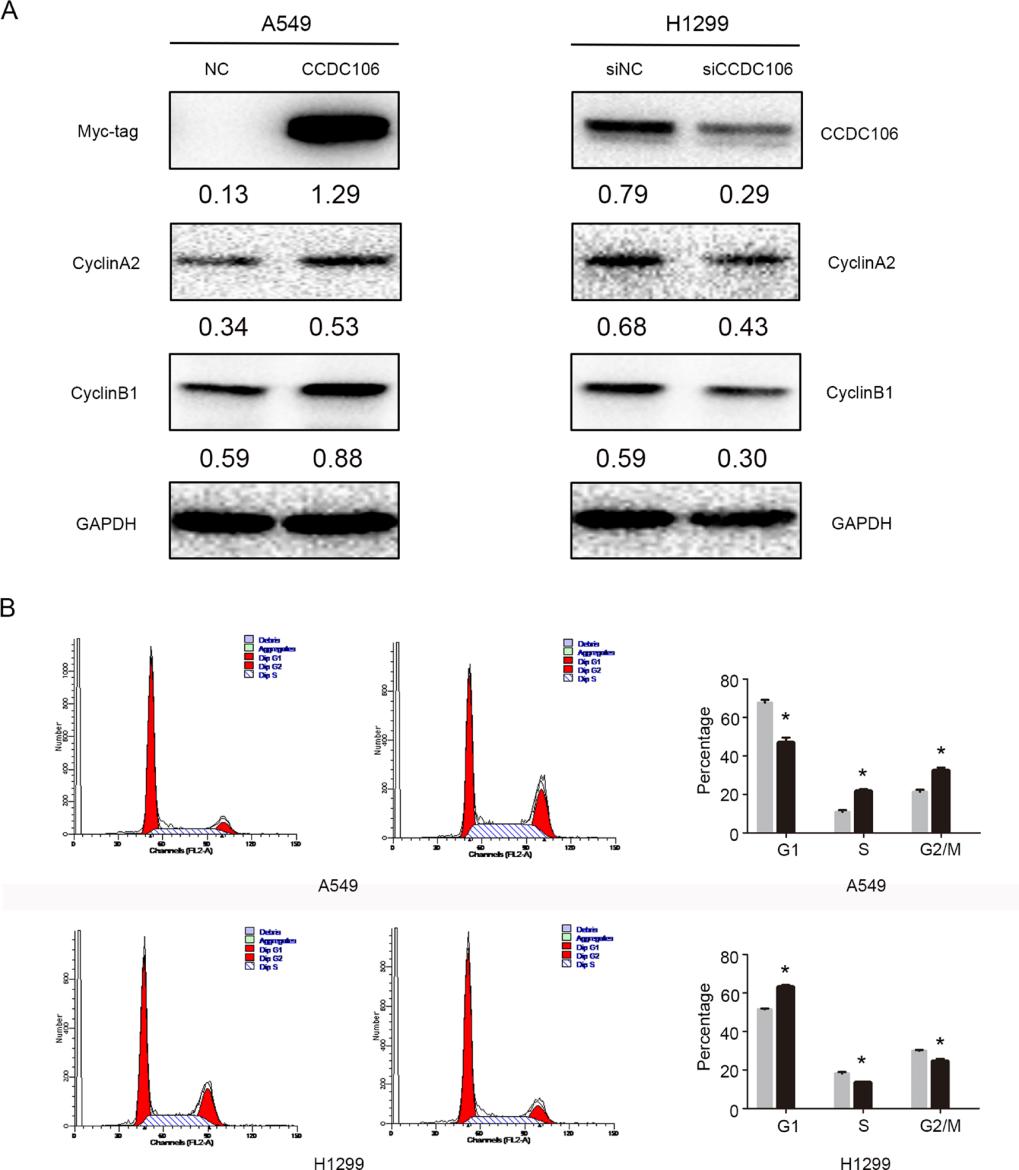
CCDC106 upregulated Cyclin A2 and Cyclin B1 Cyclin A2 and Cyclin B1 levels increased following CCDC106 overexpression, and decreased following knockdown (**A**) G1 phase was decreased, and S and G2/M phases were increased in CCDC106-overexpressing A549 cells G1 phase was increased, and S and G2/M phases were decreased in CCDC106-silenced H1299 cells (**B**).

We overexpressed or silenced CCDC106 to investigate its influence on lung cancer cell cycle regulators, including Cyclins A2, B1, D1, D2, D3, E1, E2, and H. Cyclin A2 and Cyclin B1 protein levels increased following CCDC106 overexpression, and decreased following CCDC106 knockdown (Figure 3A). No changes were observed in other cell cycle regulators (Supplementary Figure 1). Cell cycle analysis was employed to characterize A549 or H1299 cells following CCDC106 overexpression or knockdown. CCDC106 overexpression decreased the number of A549 cells in G1 phase, and increased those in S phase and G2/M phase. Correspondingly CCDC106 knockdown increased the number of H1299 cells in G1 phase, and decreased those in S phase and G2/M phase (Figure 3B).

### CCDC106 promoted AKT phosphorylation at Ser473

**Figure 4:**
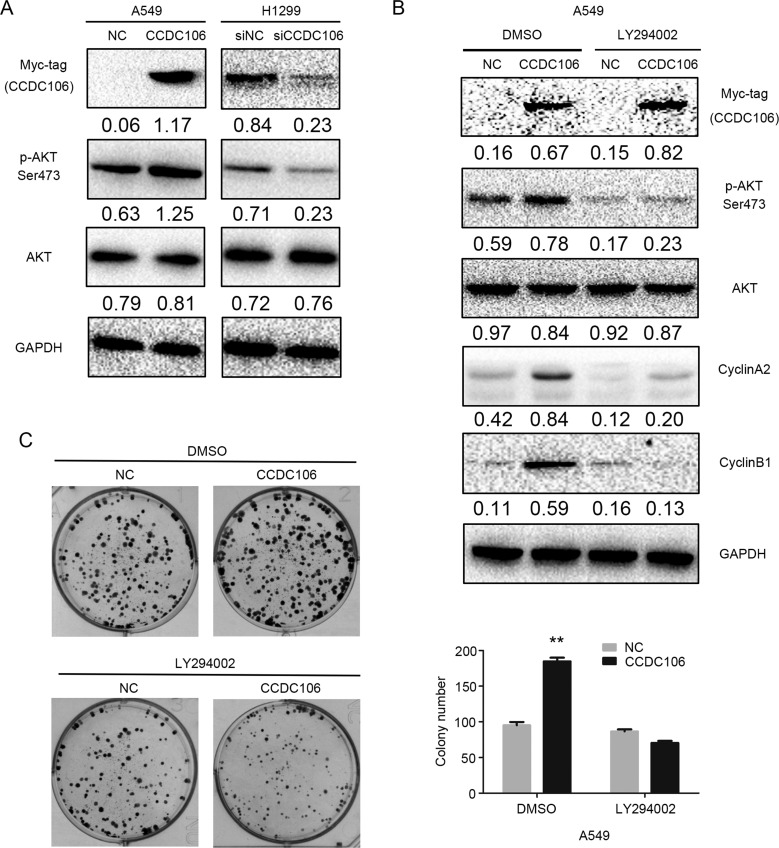
CCDC106 increased Cyclin A2 and Cyclin B1 expression via AKT activation Phosphorylated AKT (Ser473) was increased by CCDC106 overexpression in A549 cells, and decreased by CCDC106 knockdown in H1299 cells (**A**) LY294002 treatment rescued CCDC106-induced Cyclin A2 and Cyclin B1 upregulation (**B**) and NSCLC cell colony formation (**C**) **P < 0.01.

Finally, we screened key signaling pathway proteins involved in promoting cell cycle progression. WB results demonstrated that CCDC106 overexpression in A549 cells upregulated AKT phosphorylation at Ser 473; this phosphorylation was downregulated in H1299 cells following CCDC106 knockdown (Figure 4A). No other key proteins studied showed changes following CCDC106 up- or downregulation (Supplementary Figure 2). To assess whether AKT phosphorylation induced Cyclin A2 and Cyclin B1 upregulation, we treated CCDC106-overexpressing A549 cells with the AKT inhibitor, LY294002 (10 μM). Cyclin A2 and Cyclin B1 were no longer increased after LY294002 treatment (Figure 4B), and cell colony formation was reduced (Figure 4C).

## DISCUSSION

CCDC106 is a newly discovered protein, whose expression patterns and role in tumor progression were previously unknown. We found that CCDC106 was highly expressed in the cytoplasm of NSCLC tumor cells and lung cancer cell lines. Cytosolic CCDC106 overexpression correlated with advanced TNM stage, positive lymph node metastasis, and unfavorable prognosis, but not with patient age, sex, tumor differentiation, or histopathological type. Statistical analyses revealed no correlations between CCDC106 expression (positive or negative) and tumor differentiation grade in either adenocarcinomas (*P* = 0.45) or squamous cell carcinomas (*P* = 0.37) (Supplementary Table 1). We also analyzed correlations between CCDC106 expression and driver mutations, such as EGFR and KRAS, in adenocarcinoma tissues. Among 115 adenocarcinoma cases, only 38 harbored a KRAS mutation, and 73 had an EGFR mutation. No KRAS-mutated cases were also CCDC106-positive, and our results indicated that CCDC106 expression was not correlated with EGFR status (*P* = 0.825, Supplementary Table 2).

Zhou, *et al*. confirmed that CCDC106 is highly expressed in H1299 cells [2], which was consistent with our findings, and indicated that CCDC106 localized in both the cytoplasm and nucleus in Hela cells. However, our results indicated that CCDC106 localized mainly in the cytoplasm of NSCLC cells. These different CCDC106 subcellular localizations may result from different cell types or tumor origins.

Prior studies indicated that CCDC106 co-localized with p53 in Hela cells, and promoted p53 degradation. Our *in vitro* and *in vivo* studies suggested that CCDC106 overexpression increased cell proliferation, in agreement with Zhou, *et al*. [2]. We also found that CCDC106 upregulated expression of cell cycle-related proteins, such as Cyclin A2 and Cyclin B1, which regulate G2/M phase of the cell cycle [10–13]. The A549 and H1299 cell lines employed in this study harbor a wild-type *TP53* and deletion mutation, respectively. However, the function of CCDC106 in regulating proliferation or cell cycle status was similar in these lines, indicating that CCDC106-induced cell cycle and cell proliferation changes are independent of *TP53* status. Similarly, neither p53 nor phosphorylated p53 was affected by CCDC106 overexpression (Supplementary Figure 3A). Co-immunoprecipitation (co-IP) results suggested no protein interaction between CCDC106 and wild-type p53 (Supplementary Figure 3B). This was inconsistent with the findings of Zhou, et al. [2], possibly due to tissue specificity. Moreover, immunofluorescence staining showed that p53 was mainly located in the nuclei with extremely weak cytoplasmic expression, while CCDC106 was mainly located in the cytoplasm. Only dim cytosolic CCDC106-p53 co-localization was observed in A549 cells (Supplementary Figure 3C). Co-IP and immunofluorescence results suggested that CCDC106 neither binds nor degrades p53 in NSCLC cells.

We screened key signalling pathway proteins that regulate cell cycle progression and cell growth, such as MAPK and AKT pathway members [13–16]. CCDC106 overexpression activated AKT phosphorylation at Ser 473. The AKT inhibitor, LY294002, reduced Cyclin A2 and Cyclin B1 levels, as well as cell proliferation (Figure 4B–4C), indicating that p-AKT likely functions as an upstream effector of these proteins.

In conclusion, we demonstrated that CCDC106 is highly expressed and primarily localized in the cytoplasm in NSCLC tissues and lung cancer cell lines. CCDC106 cytosolic localization was correlated with advanced TNM stage, lymph node metastasis, and unfavorable prognosis in NSCLC. CCDC106 facilitated AKT phosphorylation, leading to Cyclin A2 and Cyclin B2 upregulation, and ultimately enhanced proliferation of lung cancer cells. These results suggest that CCDC106 may be a novel target for lung cancer treatment.

## MATERIALS AND METHODS

### Patients and specimens

This study was approved by the institutional review board of the China Medical University. Tissue samples were obtained from 183 patients (112 males and 71 females) who underwent complete surgical excision at the First Affiliated Hospital of China Medical University between 2010 and 2012, with a diagnosis of squamous cell carcinoma, adenocarcinoma, or large cell carcinoma. No neoadjuvant radiotherapy or chemotherapy was applied before surgery, and all patients received standard chemotherapy after surgery. Follow-up data was available for all cases. Patient survival was defined as the time from the day of surgery to the end of follow-up or the day of death due to recurrence or metastasis. Median patient age was 61 years (range: 29–80 years). Seventy-three patients were older than 61 years.

Histological examination was performed on formalin-fixed tissues, and tumors were diagnosed and classified according to the 2015 World Health Organization (WHO) classification [17] as follows: 115 adenocarcinomas, 66 squamous cell carcinomas, and two large cell carcinomas. Differentiation grade (well-differentiated: 75, moderately or poorly differentiated: 108) and pN status (pN0: 97, pN1: 29, and pN2: 57) were also recorded. Tumor staging was performed according to the seventh edition of the International Union against Cancer (UICC) TNM Staging System for Lung Cancer [18]. Fifty-one cases were pathological stage I, 69 were stage II, and 63 were stage III. Among 115 adenocarcinoma cases, 38 had a KRAS mutation, and 73 had an EGFR mutation. Among the 38 KRAS-mutated cases, none were CCDC106-positive. In contrast, 33 cases with EGFR mutation were positive (21 cases in extron 19; 12 cases in extron 21).

### Cell culture

The HBE cell line was obtained from the American Type Culture Collection (ATCC; Manassas, VA, USA). The A549, H460, H292, H1299, H661, and SK-MES-1 cell lines were obtained from the Shanghai Cell Bank (Shanghai, China). The LK2 cell line was a gift from Dr. Hiroshi Kijima (Department of Pathology and Bioscience, Hirosaki University Graduate School of Medicine, Japan). All cell lines were authenticated by short tandem repeat (STR) DNA profiling. Upon receipt, cells were frozen and individual aliquots were cultured, typically for analysis within ten passages. All cells were cultured in RPMI 1640 (Invitrogen, Carlsbad, CA, USA) supplemented with 10% fetal bovine serum (FBS; Invitrogen), 100 IU/ml penicillin (Sigma, St. Louis, MO, USA), and 100 μg/ml streptomycin (Sigma), and passaged every other day using 0.25% trypsin (Invitrogen).

### Western blotting

Total protein was extracted using lysis buffer (Pierce, Rockford, IL, USA) and quantified with the Bradford method [19]. Fifty μg total protein per sample were separated via 10% SDS-PAGE, and transferred onto polyvinylidene fluoride membranes (PVDF; Millipore, Billerica, MA, USA). Membranes were incubated overnight at 4°C with the following primary antibodies: CCDC106 (1:100, ab105354, Abcam, Cambridge, UK), GAPDH (1:5000, Sigma, St. Louis, MO, USA), Myc-tag, Cyclin A2, Cyclin B1, Cyclin D1, Cyclin D2, Cyclin D3, Cyclin E1, Cyclin E2, Cyclin H, p-P38, P38, p-ERK, ERK, p-AKT, and AKT (1:1000; Cell Signaling Technology, Danvers, MA, USA). Membranes were washed and incubated with peroxidase-conjugated anti-mouse or anti-rabbit IgG (Santa Cruz Biotechnology) at 37°C for 2 h. Bound proteins were visualized using electrochemiluminescence (Pierce, Rockford, IL, USA) and detected with a bio-imaging system (DNR Bio-Imaging Systems, Jerusalem, Israel).

### Plasmid transfection and small interfering RNAs

Plasmids pCMV6-ddk-myc and pCMV6-ddk-myc-CCDC106 were purchased from Origene (Rockville, MD, USA). CCDC106-siRNA (sc-97806) and non-coding (NC)-siRNA (sc-37007) were purchased from Santa Cruz Biotechnology. Transfection was carried out using the Lipofectamine 3000 reagent (Invitrogen) according to the manufacturer's instructions.

### MTT assay

Cells were cultured in 96-well plates in medium containing 10% fetal bovine serum at approximately 3000 cells/well 24 h after transfection. For quantitation of cell viability, cultures were stained after 4 d via MTT assay. Briefly, 20 μl of 5 mg/ml MTT (thiazolyl blue) solution was added to each well and incubated for 4 h at 37°C. Media was then removed from each well, and the resultant MTT formazan was solubilized in 150 μl of DMSO. Results were quantitated spectrophotometrically at a wavelength of 490 nm. Experiments were performed in triplicate.

### Colony formation assay

A549 and H1299 cells were transfected with pCMV6 or pCMV6-CCDC106 plasmids, negative control or CCDC106-siRNA for 48 h. Cells were then transferred into three 6-cm cell culture dishes (1000 cells per dish) and incubated for 12 d. Plates were washed with phosphate-buffered saline (PBS) and stained with Giemsa. Colonies with more than 50 cells were counted manually under a microscope. Experiments were performed in triplicate.

### Flow cytometry for cell cycle analysis

Cells (500,000) were seeded into 6-cm tissue culture dishes. Twelve h later, cells were transfected with CCDC106 overexpression plasmid or empty vector and CCDC106-siRNA or NC-siRNA. Forty-eight h after transfection, cells were harvested, fixed in 1% paraformaldehyde, washed with PBS, and stained with 5 mg/ml propidium iodide (PI) in PBS supplemented with RNase A (Roche, Indianapolis, IN) for 30 min at room temperature. Data were collected using BD systems. A one-parameter histogram was plotted according to nuclear DNA content in each cell as detected by flow cytometry. Cells in each phase of the cell cycle were determined based on DNA ploidy profiles.

### Tumor cell transplantation into nude mice

All animal studies followed the experimental animal ethics guidelines issued at China Medical University. Four-week-old female BALB/c nude mice were purchased from Slac (Shanghai, China). Mice were kept in a laminar-flow cabinet under specific pathogen-free conditions for two weeks before use. Each mouse was inoculated subcutaneously in the axilla with 5 × 10^6^ tumor cells (CCDC106-transfected A549 or corresponding vector-transfected control cells) in 0.2 mL sterile PBS. Six weeks after inoculation, mice were sacrificed and autopsied to examine tumor growth and dissemination. In addition, tumors, heart, liver, lung, and kidney were dissected. Tissues from tumors and each organ were fixed in 4% formaldehyde (Sigma) and embedded in paraffin. Serial 6-μm-thick sections were cut, stained with H&E, and examined microscopically.

### Statistical analysis

SPSS version 22.0 for Windows (SPSS, Chicago, IL, USA) was used for all analyses. The Pearson's Chi-square test and Fisher exact test were used to assess possible correlations between CCDC106 and clinicopathological factors. Kaplan-Meier survival analyses were carried out for all 183 cases and compared using the log-rank test. All of the clinicopathological parameters were included in the Cox regression model and tested by univariate analysis and multivariate analysis using the enter method. Mann-Whitney *U* test was used for western blot image analysis and invasion assay results. *P* < 0.05 was considered statistically significant.

## SUPPLEMENTARY MATERIALS FIGURES AND TABLES


