# Supplementary material for: CCDC106 promotes non-small cell lung cancer cell proliferation
Source: Oncotarget. 2017 Mar 1;8(16):26662–70. doi: 10.18632/oncotarget.15792 (PMC5432287; doi:10.18632/oncotarget.15792)
Supplement: Supplementary file 1 [file oncotarget-08-26662-s001.pdf]

CCDC106 promotes non-small cell lung cancer cell proliferation

Supplementary Materials

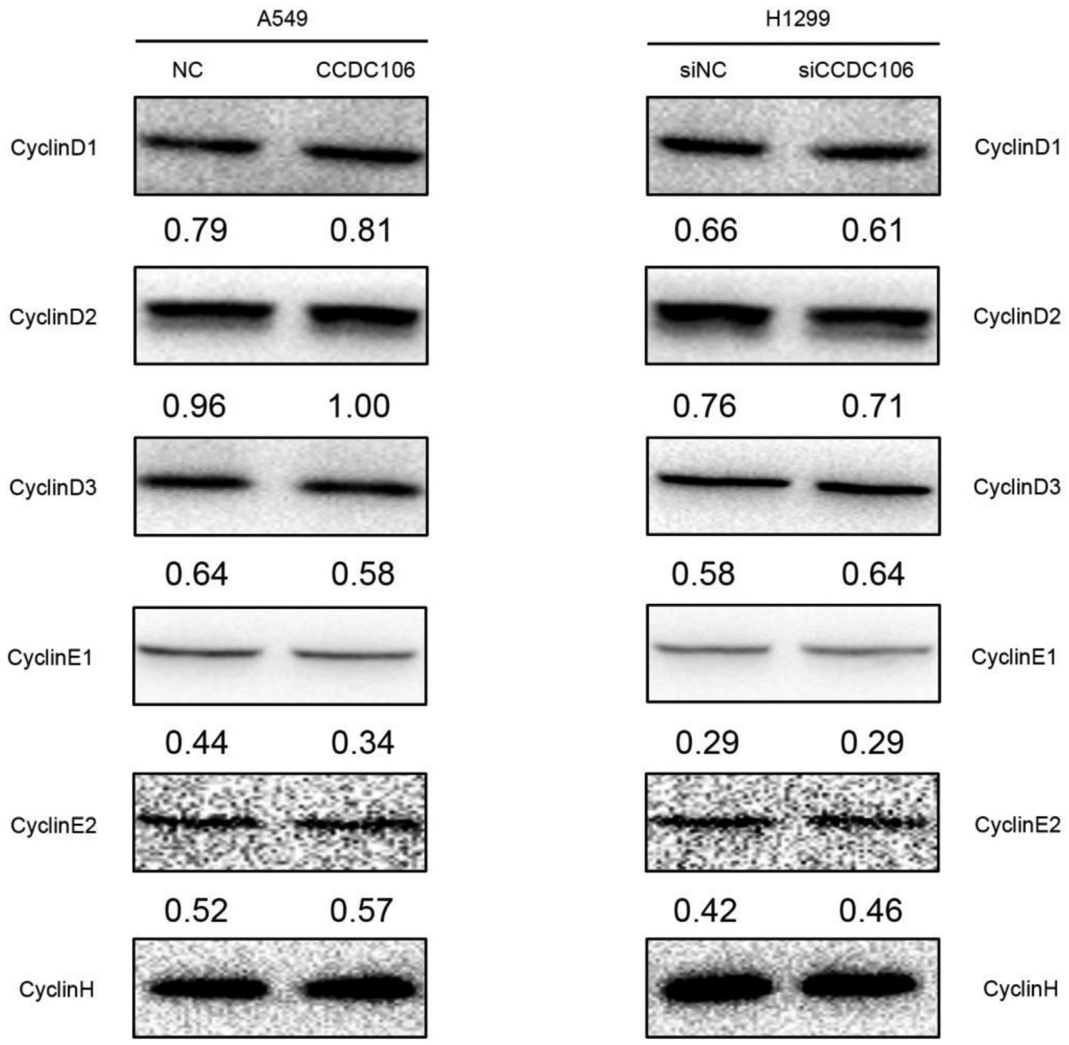

Supplementary Figure 1: The effect on cyclin proteins by overexpressing or depleting CCDC106 . When we overexpressed or interfered CCDC106 by siRNA in A549 or H1299 cells, the cyclin proteins revealed no visible changes.

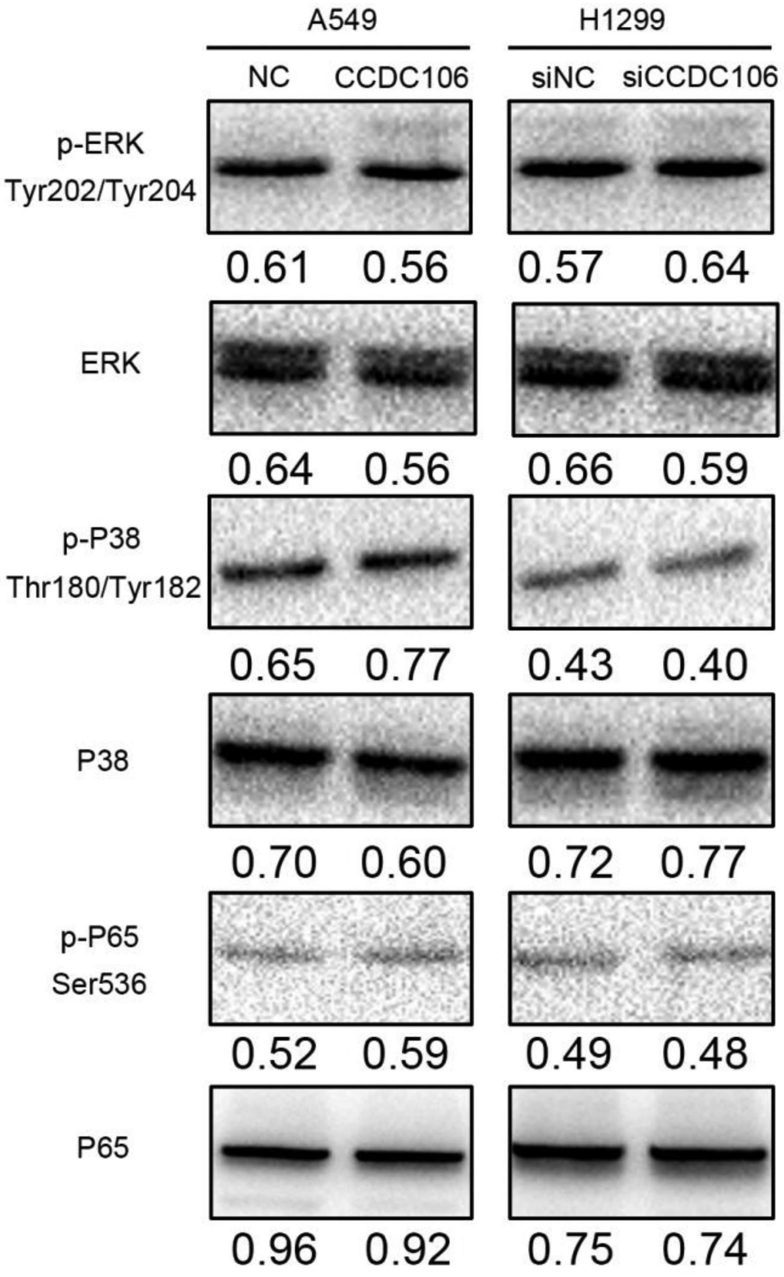

Supplementary Figure 2: The effect on signaling pathway proteins by overexpressing or depleting CCDC106. The expression of key signaling pathway proteins did not alter when we overexpressed or depleted CCDC106 in A549 or H1299 cells.

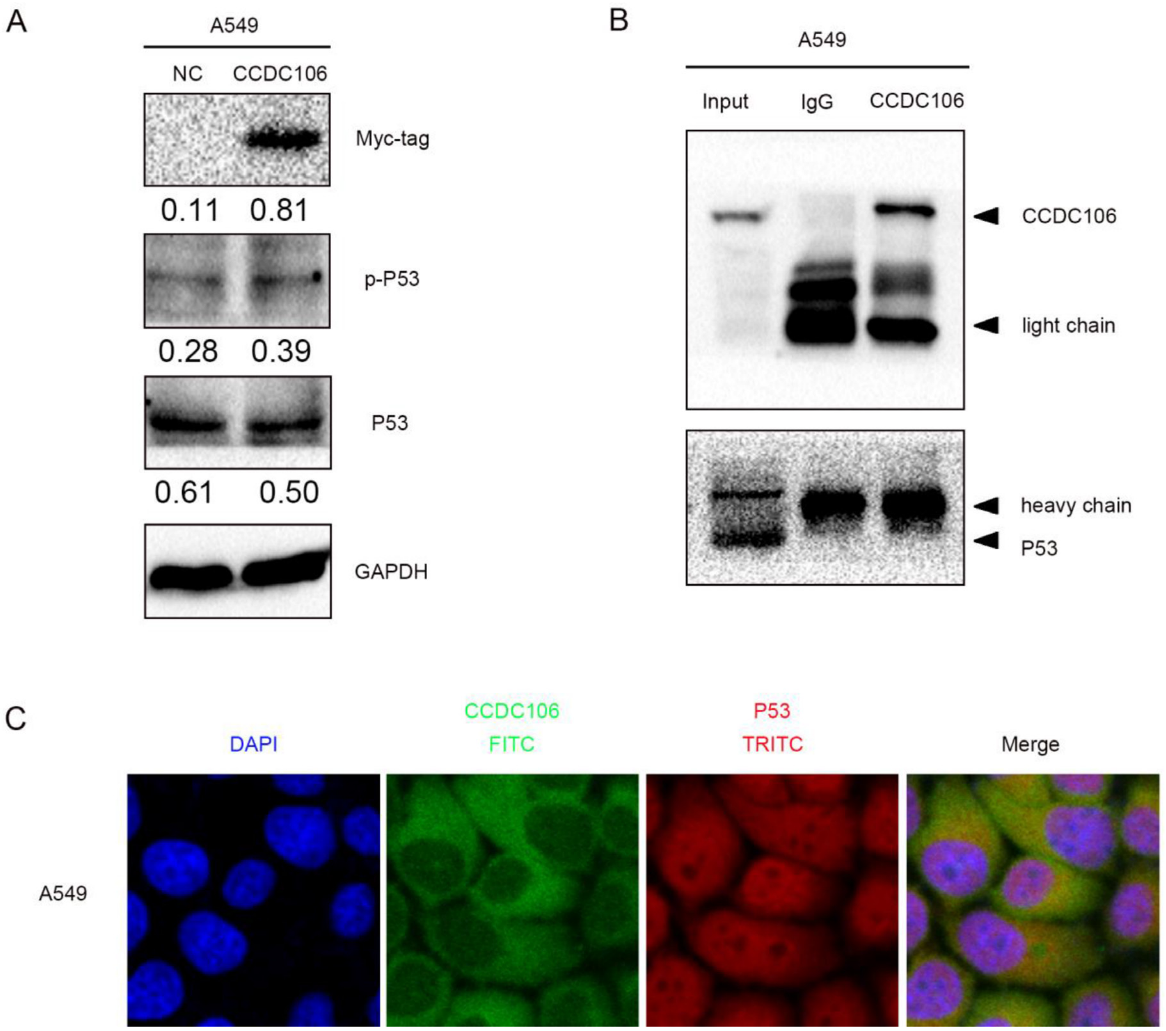

Supplementary Figure 3: The interaction between CCDC106 and P53. (**A**) When we overexpressed CCDC106 in A549 cells which harboured wild-type P53, the expression of P53 and phosphorylated P53 showed no obvious changes. (**B**) Co-IP assay was performed in A549 cells, it revealed that CCDC106 may not interacted with P53. (**C**) Immunofluorescence assay was assessed in A549 cells, the results suggested that CCDC106 localized mainly in the cytoplasm, however, P53 localized in both cytoplasm and nucleus. CCDC106 may co-localize in the cytoplasm of A549 cells.

Supplementary Table 1: The correlation between CCDC106 expression and different hisopathology type of NSCLC

Clinicopathological factors	*N*	CCDC106 Positive	CCDC106 Negative	χ2	*P*
Adenocarcinoma differentiation					
Well	59	32	27	0.807	0.45
Moderate + Poor	56	35	21		
Squamous cell carcinoma differentiation					
Well	14	5	9	1.16	0.37
Moderate + Poor	52	27	25		

Supplementary Table 2: Correlation between CCDC106 expression and EGFR status in 73 cases NSCLC

Clinicopathological factors	*N*	CCDC106 Positive	CCDC106 Negative	χ2	*P*
EGFR WT	40	22	18	0.049	0.825
EGFR Mutant	33	19	14		
